# The genome sequence of a soldierfly, *Nemotelus nigrinus *(Fallén, 1817)

**DOI:** 10.12688/wellcomeopenres.19534.1

**Published:** 2023-06-12

**Authors:** Olga Sivell, Ryan Mitchell, Duncan Sivell, Natural History Museum Genome Acquisition Lab, Darwin Tree of Life Barcoding collective, Wellcome Sanger Institute Tree of Life programme, Wellcome Sanger Institute Scientific Operations: DNA Pipelines collective, Tree of Life Core Informatics collective, Darwin Tree of Life Consortium

**Affiliations:** 1 Natural History Museum, London, England, UK

**Keywords:** Nemotelus nigrinus, a soldierfly, genome sequence, chromosomal, Diptera

## Abstract

We present a genome assembly from an individual female *Nemotelus nigrinus* (a soldierfly; Arthropoda; Insecta; Diptera; Stratiomyidae). The genome sequence is 722.2 megabases in span. Most of the assembly is scaffolded into 5 chromosomal pseudomolecules, including the X sex chromosome. The mitochondrial genome has also been assembled and is 18.94 kilobases in length.

## Species taxonomy

Eukaryota; Metazoa; Eumetazoa; Bilateria; Protostomia; Ecdysozoa; Panarthropoda; Arthropoda; Mandibulata; Pancrustacea; Hexapoda; Insecta; Dicondylia; Pterygota; Neoptera; Endopterygota; Diptera; Brachycera; Stratiomyomorpha; Stratiomyidae; Nemotelinae; Nemotelus (Fallén, 1817) (NCBI:txid343700).

## Background

*Nemotelus nigrinus* is a small black fly (body length 3.25 to 4.5 mm) from the family Stratiomyidae (Diptera), commonly called soldierflies. It has a conical face characteristic for the genus, and is entirely black except for white halteres, and yellow parts of the legs (tips of femora, anterior and middle tibiae, parts of hind tibiae, tarsi).

The species is common in southern England, the East Midlands and the coastal belt of Wales, but less common in northern England, scarce in south-east Scotland, and there are a few scattered records from Ireland ( Stubbs & Drake, 2014). The species occurs in fens and marsh on chalk and limestone, coastal marshes and around pools in the slacks of calcareous dunes. The adults are on the wing from May to August, with a peak in late June to early July ( Stubbs & Drake, 2014).

The larva and puparium have not been described. The known *Nemotelus* larvae are associated with mud or surface of standing water ( Rozkošný, 1982). A specimen of *Nemotelus nigrinus* has been reared from a swan’s nest ( Stubbs & Drake, 2014).

The high-quality genome of *Nemotelus nigrinus* was sequenced as part of the Darwin Tree of Life Project, a collaborative effort to sequence all named eukaryotic species in the Atlantic Archipelago of Britain and Ireland. Here we present a chromosomally complete genome sequence for *Nemotelus nigrinus*, based on one female specimen from Cothill Fen National Nature Reserve, England.

## Genome sequence report

**Figure 1.**
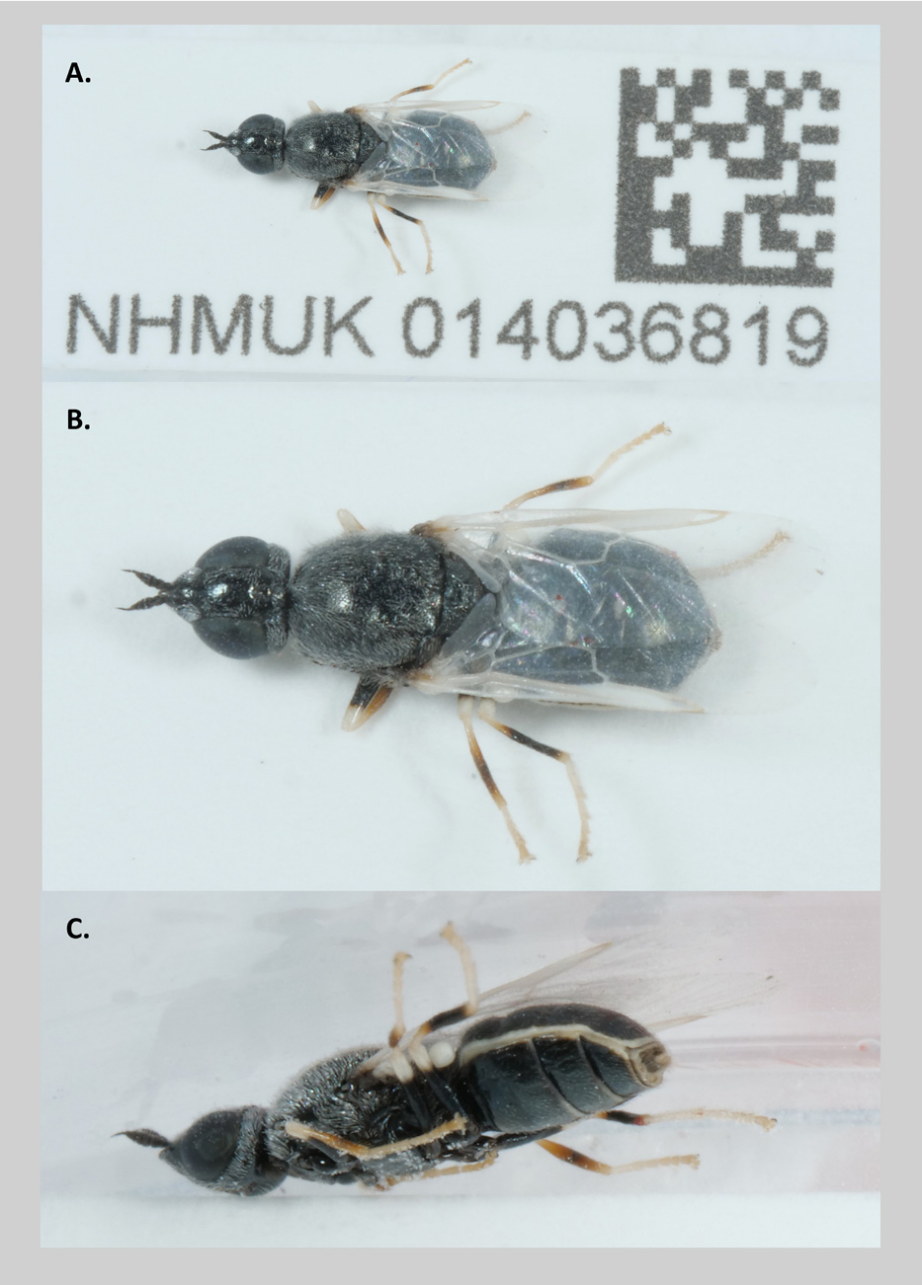
Photographs of the *Nemotelus nigrinus* (specimen NHMUK014036819, individual idNemNigr1) taken during sample preservation and processing. **A** and **B**. A habitus of the specimen in dorsal view. **C**. The specimen in lateral view.

The genome was sequenced from one female *Nemotelus nigrinus* ( Figure 1) collected from Cothill Fen National Nature Reserve (51.69, –1.33). A total of 40-fold coverage in Pacific Biosciences single-molecule HiFi long was generated. Primary assembly contigs were scaffolded with chromosome conformation Hi-C data. Manual assembly curation corrected 1,292 missing joins or mis-joins and removed 20 haplotypic duplications, reducing the assembly length by 0.62% and the scaffold number by 63.16%, and increasing the scaffold N50 by 181%.

**Figure 2.**
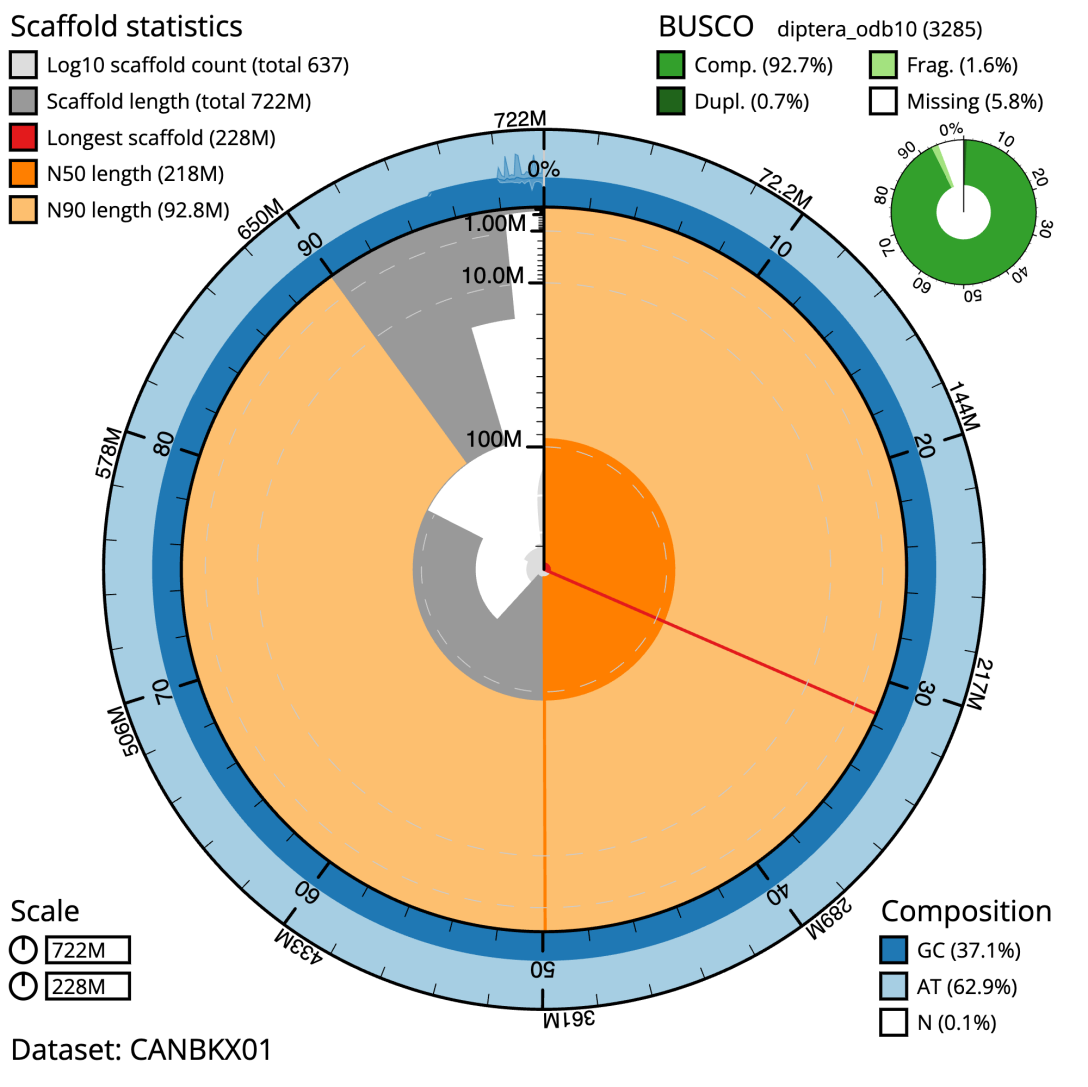
Genome assembly of *Nemotelus nigrinus*, idNemNigr1.1: metrics. The BlobToolKit Snailplot shows N50 metrics and BUSCO gene completeness. The main plot is divided into 1,000 size-ordered bins around the circumference with each bin representing 0.1% of the 722,201,843 bp assembly. The distribution of scaffold lengths is shown in dark grey with the plot radius scaled to the longest scaffold present in the assembly (228,165,927 bp, shown in red). Orange and pale-orange arcs show the N50 and N90 scaffold lengths (217,608,999 and 92,825,783 bp), respectively. The pale grey spiral shows the cumulative scaffold count on a log scale with white scale lines showing successive orders of magnitude. The blue and pale-blue area around the outside of the plot shows the distribution of GC, AT and N percentages in the same bins as the inner plot. A summary of complete, fragmented, duplicated and missing BUSCO genes in the diptera_odb10 set is shown in the top right. An interactive version of this figure is available at https://blobtoolkit.genomehubs.org/view/idNemNigr1.1/dataset/CANBKX01/snail.

The final assembly has a total length of 722.2 Mb in 636 sequence scaffolds with a scaffold N50 of 217.6 Mb ( Table 1). Most (98.15%) of the assembly sequence was assigned to 5 chromosomal-level scaffolds, representing 4 autosomes and the X sex chromosome. Chromosome-scale scaffolds confirmed by the Hi-C data are named in order of size ( Figure 2– Figure 5; Table 2). There are regions of unknown order and orientation in the following positions: Chromosome 1 (82,000 to 152,000 kbp, Chromosome 2 (100,000 to 135,000 kbp), Chromosome 3 (72,400 to 97,000 kbp) and Chromosome 4 (65,000 to 68,500 kbp). While not fully phased, the assembly deposited is of one haplotype. Contigs corresponding to the second haplotype have also been deposited. The mitochondrial genome was also assembled and can be found as a contig within the multifasta file of the genome submission.

**Table 1.** Genome data for *Nemotelus nigrinus*, idNemNigr1.1.

Project accession data		
Assembly identifier	idNemNigr1.1	
Species	*Nemotelus nigrinus*	
Specimen	idNemNigr1	
NCBI taxonomy ID	343700	
BioProject	PRJEB55137	
BioSample ID	SAMEA11025071	
Isolate information	idNemNigr1, female (DNA sequencing and Hi-C scaffolding)	
Assembly metrics *		*Benchmark*
Consensus quality (QV)	56.4	*≥ 50*
*k*-mer completeness	99.99%	*≥ 95%*
BUSCO **	C:92.7%[S:92.0%,D:0.7%], F:1.6%,M:5.8%,n:3,285	*C ≥ 95%*
Percentage of assembly mapped to chromosomes	98.15%	*≥ 95%*
Sex chromosomes	X chromosome	*localised homologous pairs*
Organelles	Mitochondrial genome assembled	*complete single alleles*
Raw data accessions		
PacificBiosciences SEQUEL II	ERR10033484	
Hi-C Illumina	ERR10038432	
Genome assembly		
Assembly accession	GCA_947369275.1	
*Accession of alternate haplotype*	GCA_947369265.1	
Span (Mb)	722.2	
Number of contigs	3,490	
Contig N50 length (Mb)	1.6	
Number of scaffolds	637	
Scaffold N50 length (Mb)	217.6	
Longest scaffold (Mb)	228.2	

* Assembly metric benchmarks are adapted from column VGP-2020 of “Table 1: Proposed standards and metrics for defining genome assembly quality” from ( Rhie *et al.*, 2021). ** BUSCO scores based on the diptera_odb10 BUSCO set using v5.3.2. C = complete [S = single copy, D = duplicated], F = fragmented, M = missing, n = number of orthologues in comparison. A full set of BUSCO scores is available at https://blobtoolkit.genomehubs.org/view/idNemNigr1.1/dataset/CANBKX01/busco.

**Figure 3.**
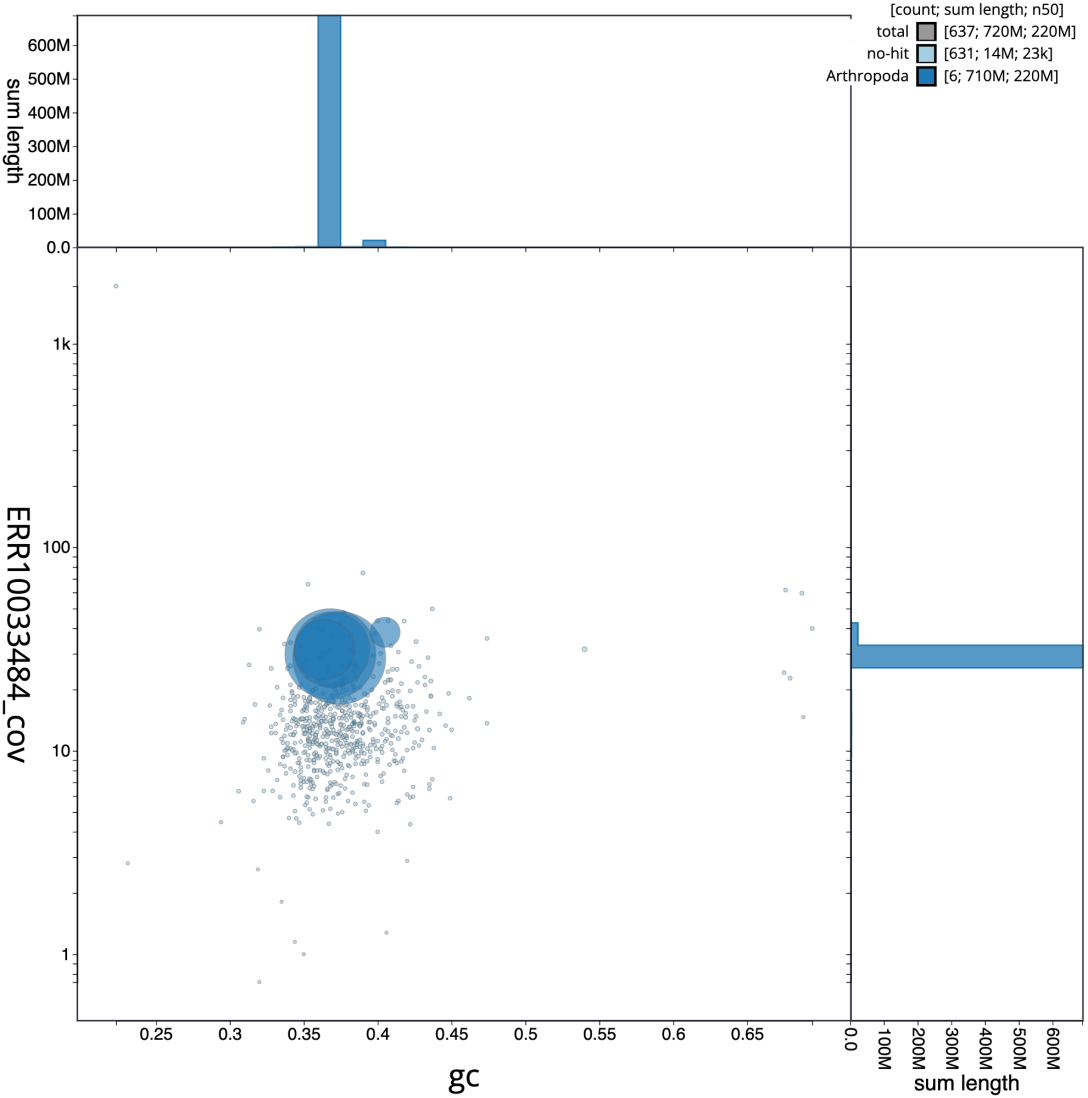
Genome assembly of *Nemotelus nigrinus*, idNemNigr1.1: BlobToolKit GC-coverage plot. Scaffolds are coloured by phylum. Circles are sized in proportion to scaffold length. Histograms show the distribution of scaffold length sum along each axis. An interactive version of this figure is available at https://blobtoolkit.genomehubs.org/view/idNemNigr1.1/dataset/CANBKX01/blob.

**Figure 4.**
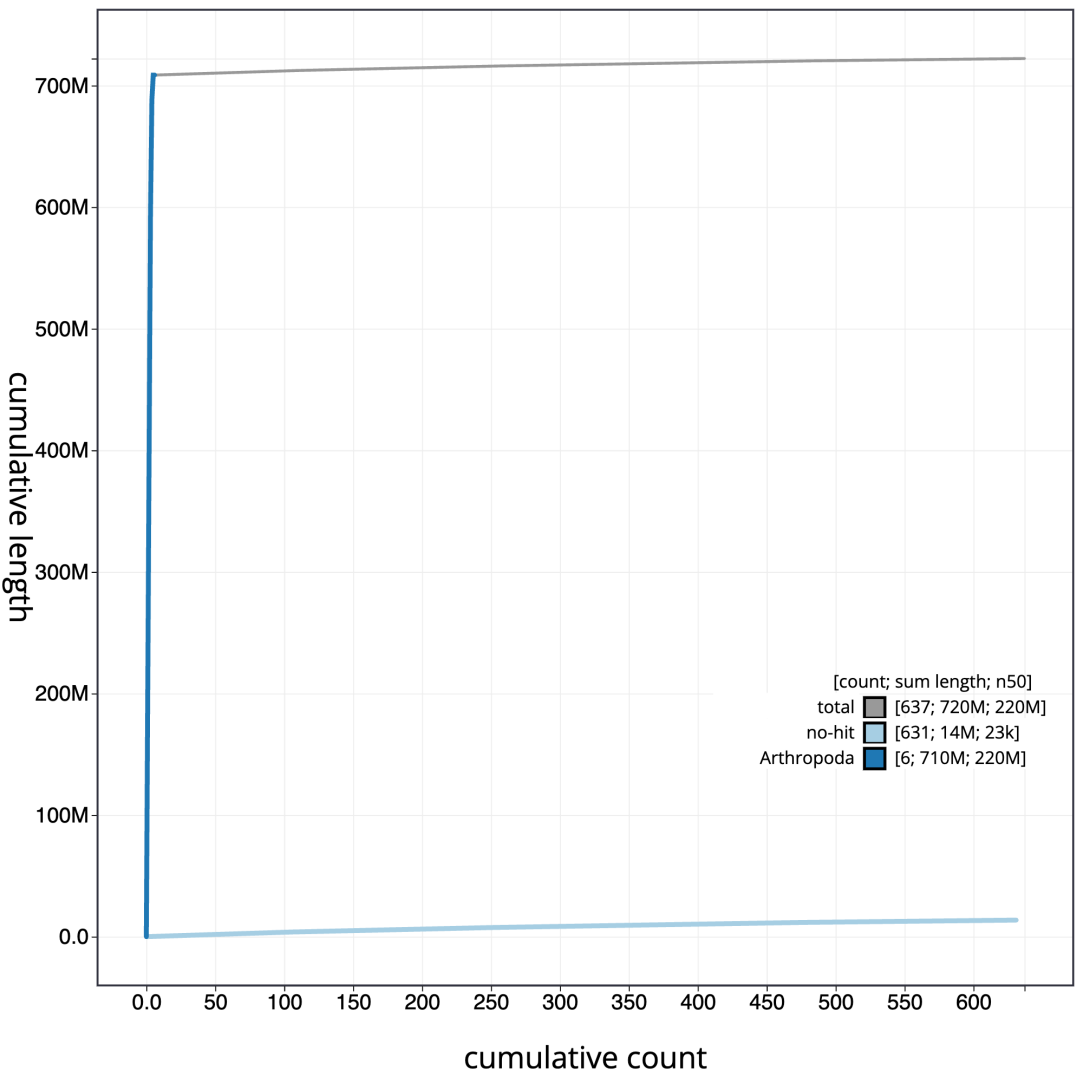
Genome assembly of *Nemotelus nigrinus*, idNemNigr1.1: BlobToolKit cumulative sequence plot. The grey line shows cumulative length for all scaffolds. Coloured lines show cumulative lengths of scaffolds assigned to each phylum using the buscogenes taxrule. An interactive version of this figure is available at https://blobtoolkit.genomehubs.org/view/idNemNigr1.1/dataset/CANBKX01/cumulative.

**Figure 5.**
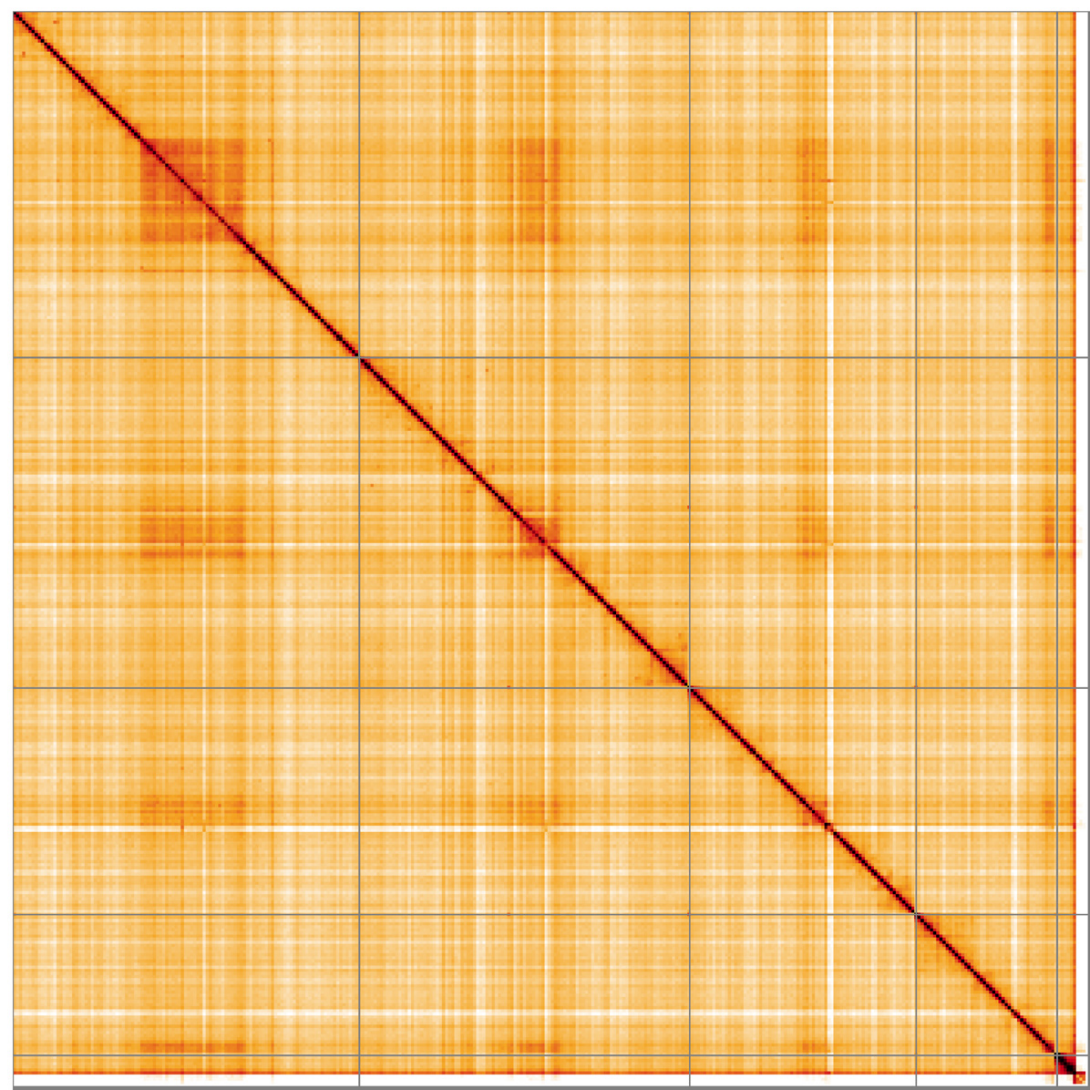
Genome assembly of *Nemotelus nigrinus*, idNemNigr1.1: Hi-C contact map of the idNemNigr1.1 assembly, visualised using HiGlass. Chromosomes are shown in order of size from left to right and top to bottom. An interactive version of this figure may be viewed at https://genome-note-higlass.tol.sanger.ac.uk/l/?d=RJt7Caf-RHia-GX3chzEJA.

**Table 2.** Chromosomal pseudomolecules in the genome assembly of *Nemotelus nigrinus*, idNemNigr1.

INSDC accession	Chromosome	Length (Mb)	GC%
OX376372.1	1	228.17	37.5
OX376373.1	2	217.61	37.0
OX376374.1	3	149.27	37.0
OX376375.1	4	92.83	36.5
OX376376.1	X	20.7	40.5
OX376377.1	MT	0.02	22.5

The estimated Quality Value (QV) of the final assembly is 56.4 with *k*-mer completeness of 99.99%, and the assembly has a BUSCO v5.3.2 completeness of 92.7% (single = 92.0%, duplicated = 0.7%), using the diptera_odb10 reference set ( *n* = 3,285).

Metadata for specimens, spectral estimates, sequencing runs, contaminants and pre-curation assembly statistics can be found at https://links.tol.sanger.ac.uk/species/343700.

## Methods

### Sample acquisition and nucleic acid extraction

A female of *Nemotelus nigrinus* (idNemNigr1, NHMUK014036819) was swept using an insect net from the vegetation in Cothill Fen National Nature Reserve, England (51.69, –1.329) on 2021-06-19. The collector was Olga Sivell (Natural History Museum). The specimen was identified by Duncan Sivell (Natural History Museum) using Stubbs and Drake (2014). The specimen was snap-frozen using dry ice. The tissue samples taken from it were stored in a CoolRack prior to genome sequencing.

DNA was extracted at the Tree of Life laboratory, Wellcome Sanger Institute (WSI). The idNemNigr1 specimen was weighed and dissected on dry ice with tissue set aside for Hi-C sequencing. Whole organism tissue was disrupted using a Nippi Powermasher fitted with a BioMasher pestle. High molecular weight (HMW) DNA was extracted using the Qiagen MagAttract HMW DNA extraction kit. HMW DNA was sheared into an average fragment size of 12–20 kb in a Megaruptor 3 system with speed setting 30. Sheared DNA was purified by solid-phase reversible immobilisation using AMPure PB beads with a 1.8X ratio of beads to sample to remove the shorter fragments and concentrate the DNA sample. The concentration of the sheared and purified DNA was assessed using a Nanodrop spectrophotometer and Qubit Fluorometer and Qubit dsDNA High Sensitivity Assay kit. Fragment size distribution was evaluated by running the sample on the FemtoPulse system.

### Sequencing

Pacific Biosciences HiFi circular consensus DNA sequencing libraries were constructed according to the manufacturers’ instructions. DNA sequencing was performed by the Scientific Operations core at the WSI on the Pacific Biosciences SEQUEL II (HiFi) instrument. Hi-C data were also generated from tissue of idNemNigr1 using the Arima2 kit and sequenced on the Illumina NovaSeq 6000 instrument.

### Genome assembly, curation and evaluation

Assembly was carried out with Hifiasm ( Cheng *et al.*, 2021) and haplotypic duplication was identified and removed with purge_dups ( Guan *et al.*, 2020). The assembly was then scaffolded with Hi-C data ( Rao *et al.*, 2014) using YaHS ( Zhou *et al.*, 2023). The assembly was checked for contamination and corrected as described previously ( Howe *et al.*, 2021). Manual curation was performed using HiGlass ( Kerpedjiev *et al.*, 2018) and Pretext ( Harry, 2022). The mitochondrial genome was assembled using MitoHiFi ( Uliano-Silva *et al.*, 2022), which runs MitoFinder ( Allio *et al.*, 2020) or MITOS ( Bernt *et al.*, 2013) and uses these annotations to select the final mitochondrial contig and to ensure the general quality of the sequence.

A Hi-C map for the final assembly was produced using bwa-mem2 ( Vasimuddin *et al.*, 2019) in the Cooler file format ( Abdennur & Mirny, 2020). To assess the assembly metrics, the *k*-mer completeness and QV consensus quality values were calculated in Merqury ( Rhie *et al.*, 2020). This work was done using Nextflow ( Di Tommaso *et al.*, 2017) DSL2 pipelines “sanger-tol/readmapping” ( Surana *et al.,* 2023a) and “sanger-tol/genomenote” ( Surana *et al.,* 2023b). The genome was analysed within the BlobToolKit environment ( Challis *et al.*, 2020) and BUSCO scores ( Manni *et al.*, 2021; Simão *et al.*, 2015) were calculated.

Table 3 contains a list of relevant software tool versions and sources.

**Table 3.** Software tools: versions and sources.

Software tool	Version	Source
BlobToolKit	4.1.5	https://github.com/blobtoolkit/blobtoolkit
BUSCO	5.3.2	https://gitlab.com/ezlab/busco
Hifiasm	0.16.1-r375	https://github.com/chhylp123/hifiasm
HiGlass	1.11.6	https://github.com/higlass/higlass
Merqury	MerquryFK	https://github.com/thegenemyers/MERQURY.FK
MitoHiFi	2	https://github.com/marcelauliano/MitoHiFi
PretextView	0.2	https://github.com/wtsi-hpag/PretextView
purge_dups	1.2.3	https://github.com/dfguan/purge_dups
sanger-tol/genomenote	v1.0	https://github.com/sanger-tol/genomenote
sanger-tol/readmapping	1.1.0	https://github.com/sanger-tol/readmapping/tree/1.1.0
YaHS	yahs-1.1.91eebc2	https://github.com/c-zhou/yahs

### Wellcome Sanger Institute – Legal and Governance

The materials that have contributed to this genome note have been supplied by a Darwin Tree of Life Partner.

The submission of materials by a Darwin Tree of Life Partner is subject to the **‘Darwin Tree of Life Project Sampling Code of Practice’**, which can be found in full on the Darwin Tree of Life website here. By agreeing with and signing up to the Sampling Code of Practice, the Darwin Tree of Life Partner agrees they will meet the legal and ethical requirements and standards set out within this document in respect of all samples acquired for, and supplied to, the Darwin Tree of Life Project.

Further, the Wellcome Sanger Institute employs a process whereby due diligence is carried out proportionate to the nature of the materials themselves, and the circumstances under which they have been/are to be collected and provided for use. The purpose of this is to address and mitigate any potential legal and/or ethical implications of receipt and use of the materials as part of the research project, and to ensure that in doing so we align with best practice wherever possible.

The overarching areas of consideration are:

- Ethical review of provenance and sourcing of the material
- Legality of collection, transfer and use (national and international)

Each transfer of samples is further undertaken according to a Research Collaboration Agreement or Material Transfer Agreement entered into by the Darwin Tree of Life Partner, Genome Research Limited (operating as the Wellcome Sanger Institute), and in some circumstances other Darwin Tree of Life collaborators.

## Data Availability

European Nucleotide Archive: *Nemotelus nigrinus*. Accession number PRJEB55137; https://identifiers.org/ena.embl/PRJEB55137. ( Wellcome Sanger Institute, 2022) The genome sequence is released openly for reuse. The *Nemotelus nigrinus* genome sequencing initiative is part of the Darwin Tree of Life (DToL) project. All raw sequence data and the assembly have been deposited in INSDC databases. The genome will be annotated using available RNA-Seq data and presented through the Ensembl pipeline at the European Bioinformatics Institute. Raw data and assembly accession identifiers are reported in Table 1.

## Author information

Members of the Natural History Museum Genome Acquisition Lab are listed here:

https://doi.org/10.5281/zenodo.4790042.

Members of the Darwin Tree of Life Barcoding collective are listed here: https://doi.org/10.5281/zenodo.4893703.

Members of the Wellcome Sanger Institute Tree of Life programme are listed here: https://doi.org/10.5281/zenodo.4783585.

Members of Wellcome Sanger Institute Scientific Operations: DNA Pipelines collective are listed here: https://doi.org/10.5281/zenodo.4790455.

Members of the Tree of Life Core Informatics collective are listed here: https://doi.org/10.5281/zenodo.5013541.

Members of the Darwin Tree of Life Consortium are listed here: https://doi.org/10.5281/zenodo.4783558.
