# Supplementary material for: The genome sequence of a soldierfly, Nemotelus nigrinus (Fallén, 1817)
Source: Wellcome Open Res. 2023 Jun 12;8:240. [Version 1] doi: 10.12688/wellcomeopenres.19534.1 (PMC11128052; doi:10.12688/wellcomeopenres.19534.1)
Supplement: Peer review history

# Reviewer report: Reviewer response for version 1

**DOI:** 10.21956/wellcomeopenres.21638.r73689

**Published:** 2024-05-25

**Authors:** Annabel Whibley

**Affiliations:** 1 Bragato Research Institute, Riversdale, Blenheim, 7201, New Zealand; 2 The University of Auckland, Auckland, Auckland, New Zealand

Sivell and colleagues report the genome assembly of the soldier fly *Nemotelus nigrinus. *This is a thorough and organised genome report, with the use of appropriate methods and clear reporting, as is typical for assemblies that have come through the Darwin Tree of Life project  pipelines.

 I have just a few minor comments:

 (1) I suggest prefacing the collection site co-ordinates with latitude and longitude.

 (2) The assembly contiguity statistics for this assembly are a little inferior to other assemblies I have seen come through this process   (i.e. there are quite a large number of scaffolds, even after considerable manual curation). Were there particular features of the HiFi library that account for this?

 (3) Do the regions of unknown order and orientation (one per autosome) share particular features/characteristics?

 (4) The Yahs pipeline (https://github.com/c-zhou/yahs)  specifies several routes to read mapping, can you specify which one was used in this case?

Are sufficient details of methods and materials provided to allow replication by others?

Yes

Is the rationale for creating the dataset(s) clearly described?

Yes

Are the datasets clearly presented in a useable and accessible format?

Yes

Are the protocols appropriate and is the work technically sound?

Yes

Reviewer Expertise:

Genomics, Evolution

I confirm that I have read this submission and believe that I have an appropriate level of expertise to confirm that it is of an acceptable scientific standard.

# Reviewer report: Reviewer response for version 1

**DOI:** 10.21956/wellcomeopenres.21638.r80586

**Published:** 2024-05-08

**Authors:** Hanno Schmidt

**Affiliations:** 1 Johannes Gutenberg University Mainz, Mainz, Germany

In the manuscript “The genome sequence of a soldierfly, *Nemotelus nigrinus* (Fallén, 1817)” by Olga Sivell *et al.* the authors present a genome assembly of a non-model organism fly. The assembly is based on PacBio HiFi reads and additional Hi-C data. The methods used are appropriate and the resulting assembly is at the chromosome level and of good quality. I would particularly like to emphasize the documentation of sampling and initial processing. This is sometimes presented a little too superficially in genome sequencing projects. Everything is traceable here, from sampling of the specimen, species identification, specimen identifiers and DNA extraction. A hyperlink provided in the manuscript leads to even more information on sample, sequencing and assembly. The text is concise, but clear and understandable. The genome data is publicly available.

Are sufficient details of methods and materials provided to allow replication by others?

Yes

Is the rationale for creating the dataset(s) clearly described?

Yes

Are the datasets clearly presented in a useable and accessible format?

Yes

Are the protocols appropriate and is the work technically sound?

Yes

Reviewer Expertise:

genomics

I confirm that I have read this submission and believe that I have an appropriate level of expertise to confirm that it is of an acceptable scientific standard.
